# Porcine Kobuvirus in Piglets, Thailand

**DOI:** 10.3201/eid1512.090724

**Published:** 2009-12

**Authors:** Pattara Khamrin, Niwat Maneekarn, Aphisek Kongkaew, Sompreeya Kongkaew, Shoko Okitsu, Hiroshi Ushijima

**Affiliations:** Aino University, Tokyo, Japan (P. Khamrin, H. Ushijima); Chiang Mai University, Chiang Mai, Thailand (N. Maneekarn, A. Kongkaew, S. Kongkaew); Aino College, Tokyo (S. Okitsu)

**Keywords:** Porcine kobuvirus, piglets, diarrhea, Thailand, viruses, letter

**To the Editor:** To date, the genus *Kobuvirus* has consisted of 2 officially recognized species, *Aichi virus* and *Bovine Kobuvirus* (*1*). Aichi virus has been shown to be associated with acute gastroenteritis in humans (*2*–*4*), and bovine kobuvirus has been detected only in cattle (*5*,*6*). Most recently, a third candidate species of *Kobuvirus* has been described in pigs by 2 different groups of investigators from Hungary and the People’s Republic of China (*7*,*8*). This new candidate species was serendipitously recognized in stool specimens from pigs when PCR products (≈1,100 bp) were amplified by using a primer pair for the detection of caliciviruses (*7*).

Nucleotide sequences of these nonspecific PCR products were similar to those of the U-1 bovine kobuvirus and Aichi virus A846/88 reference strains; sequence identities ranged from 73% to 79% at the nucleotide level and from 69% to 70% at the amino acid (*7*). The representative strain of a new candidate species of porcine kobuvirus, S-1-HUN (Porcine kobuvirus/swine/S-1-HUN/2007/Hungary), has been analyzed to determine its complete genome sequence and genetic organization (*9*). The RNA genome of the S-1-HUN strain comprises 8,210 nt, with a genome organization analogous to that of picornaviruses. Therefore, this strain is tentatively classified as a new species of the genus *Kobuvirus*, and named porcine kobuvirus (*7*,*9*).

Currently, 2 reports have described the epidemiologic feature of porcine kobuvirus in healthy piglets. Thirty-nine (65%) of 60 stool samples collected from pigs in Hungary were positive for porcine kobuvirus by reverse transcription–PCR (RT-PCR) (*9*). Another report from China found that the prevalence of porcine kobuvirus was 30% (97 of 322 piglets) (*8*). These findings suggested that porcine kobuvirus infections are common in piglets. However, whether this agent is associated with particular diseases, including gastroenteritis, in piglets was not clear.

We conducted an epidemiologic survey of porcine kobuvirus and report the detection of this virus in the stool specimens of piglets with diarrhea. Sequence and phylogenetic analyses of the porcine kobuvirus strains were carried out to determine their evolutionary relationships with kobuvirus strains previously reported.

A total of 98 stool specimens were collected from piglets with diarrhea from 6 farms in Chiang Mai Province, Thailand, during 2001–2003. Age of the piglets ranged from 7 to 49 days old. Porcine kobuvirus was detected in fecal specimens by RT-PCR (*9*). The representative strains of porcine kobuvirus detected in our study were analyzed further by direct sequencing of their PCR amplicons (216 bp) by using BigDye Terminator Cycle Sequencing Kit (Applied Biosystems, Foster City, CA, USA). Sequences of these fragments were compared with those of reference strains available in the NCBI GenBank database by using BLAST server (http://blast.ncbi.nlm.nih.gov/Blast.cgi). Phylogenetic and molecular evolutionary analyses were conducted by using MEGA 4 (*10*). Nucleotide sequences of porcine kobuvirus strains described in this study were deposited in GenBank under accession nos. GQ152093–GQ152122.

Prevalence of porcine kobuvirus was exceptionally high in piglets with diarrhea, 99% (97 of 98 specimens). Thirty representative strains of porcine kobuvirus detected in this study were randomly selected, sequenced, and analyzed to determine their evolutionary relationships with other kobuvirus reference strains. The partial 3D region among all 30 porcine kobuvirus strains was highly conserved, with nucleotide sequence identities >90%. In addition, our strains were most closely related to 2 porcine kobuvirus reference strains (S-1-HUN and Swine/2007/CHN) available in GenBank, with the nucleotide sequence identity ranging from 91.5% to 96.3%. Phylogenetic analysis of partial 3D nucleotide sequences of our porcine kobuvirus strains, together with published sequences of porcine kobuvirus reference strains (and those of Aichi virus and bovine kobuvirus), is shown in the Figure. The phylogenetic tree confirmed that all strains we identified belonged to the porcine kobuvirus species and formed a tight cluster in a monophyletic branch with the other 2 porcine kobuvirus reference strains (S-1-HUN and Swine/2007/CHN). These strains are also distantly related to standard strains of Aichi virus and bovine kobuvirus. Recently, 18 sequences of partial 3D region of the porcine kobuvirus strains detected in China have been deposited in GenBank. Unfortunately, the specific position of PCR amplification of the strains found in China was different from that of our strains (*8*). Therefore, the relationship between these strains could not be analyzed.

**Figure:**
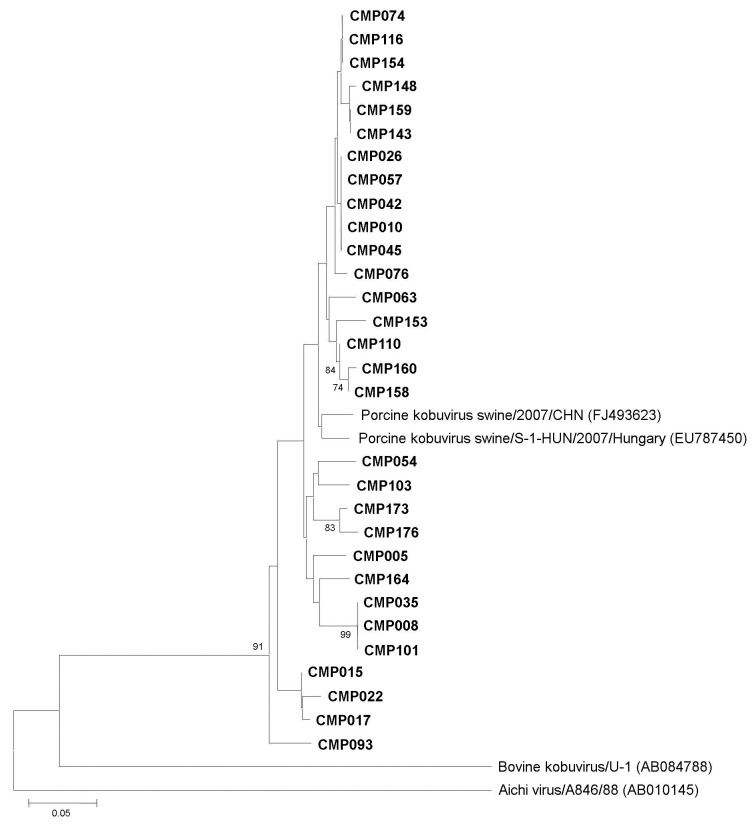
Phylogenetic analysis of the partial nucleotide sequence encoding the 3D region of porcine kobuviruses (in **boldface**) isolated in Thailand, 2001–2003, and other reference strains. The tree was generated on the basis of the neighbor-joining method by using the MEGA4 program (*10*). Scale bar indicates nucleotide substitutions per site.

Porcine kobuviruses have previously been reported only in healthy pigs (*7*–*9*). In our study, the exceptionally high prevalence of porcine kobuviruses (99%) has been observed in piglets with acute gastroenteritis; those samples were negative for rotavirus infection as determined previously by RT-PCR. However, associations of this agent with enteric diseases in pigs remains unclear because no data were available that tested for porcine kobuvirus in pigs without gastroenteritis from the farms in the same area. In addition, infection with other pathogens that may cause diarrhea in pigs, such as bacteria or other porcine caliciviruses, needs to be ruled out. Further extensive epidemiologic surveillance and comprehensive characterization of porcine kobuvirus strains from other areas may help clarify the distribution, heterogeneity, and association of porcine kobuviruses with enteric diseases in pigs.

## References

[1] Racaniello VR. Picornaviridae: The viruses and their replication. In: Knipe DM, Howley PM, editors. Fields virology, 5th ed., vol 1. Philadelphia: Lippincott Williams and Wilkins; 2007. p. 795–838.

[2] Oh DY, Silva PA, Hauroeder B, Diedrich S, Cardoso DD, Schreier E. Molecular characterization of the first Aichi viruses isolated in Europe and in South America. Arch Virol. 2006;151:1199–206. 10.1007/s00705-005-0706-7 16421634

[3] Pham NT, Khamrin P, Nguyen TA, Kanti DS, Phan TG, Okitsu S, Isolation and molecular characterization of Aichi viruses from fecal specimens collected in Japan, Bangladesh, Thailand, and Vietnam. J Clin Microbiol. 2007;45:2287–8. 10.1128/JCM.00525-07 17522267 PMC1932998

[4] Yamashita T, Sakae K, Kobayashi S, Ishihara Y, Miyake T, Mubina A, Isolation of cytopathic small round virus (Aichi virus) from Pakistani children and Japanese travelers from Southeast Asia. Microbiol Immunol. 1995;39:433–5. 8551977 10.1111/j.1348-0421.1995.tb02225.x

[5] Yamashita T, Ito M, Kabashima Y, Tsuzuki H, Fujiura A, Sakae K. Isolation and characterization of a new species of kobuvirus associated with cattle. J Gen Virol. 2003;84:3069–77. 10.1099/vir.0.19266-0 14573811

[6] Khamrin P, Maneekarn N, Peerakome S, Okitsu S, Mizuguchi M, Ushijima H. Bovine kobuviruses from cattle with diarrhea. Emerg Infect Dis. 2008;14:985–6. 10.3201/eid1406.070784 18507924 PMC2600271

[7] Reuter G, Boldizsár A, Kiss I, Pankovics P. Candidate new species of *Kobuvirus* in porcine hosts. Emerg Infect Dis. 2008;14:1968–70. 10.3201/eid1412.080797 19046542 PMC2634637

[8] Yu JM, Jin M, Zhang Q, Li HY, Li DD, Xu ZQ, Candidate porcine Kobuvirus, China. Emerg Infect Dis. 2009;15:823–5. 10.3201/eid1505.081518 19402982 PMC2687011

[9] Reuter G, Boldizsár A, Pankovics P. Complete nucleotide and amino acid sequences and genetic organization of porcine kobuvirus, a member of a new species in the genus *Kobuvirus*, family *Picornaviridae.* Arch Virol. 2009;154:101–8. 10.1007/s00705-008-0288-2 19096904

[10] Tamura K, Dudley J, Nei M, Kumar S. MEGA4: molecular evolutionary genetics analysis (MEGA) software version 4.0. Mol Biol Evol. 2007;24:1596–9. 10.1093/molbev/msm092 17488738

